# Cultural variations in sentiments

**DOI:** 10.1186/2193-1801-3-170

**Published:** 2014-04-01

**Authors:** David R Heise

**Affiliations:** Department of Sociology, Indiana University, Indiana, USA

**Keywords:** Culture, Affective meanings, Emotions

## Abstract

The largest in-depth cross-cultural study of the 20th Century, directed by psychologist Charles Osgood at the University of Illinois, demonstrated that the affective meanings of concepts vary along three dimensions within all 30 cultures considered in the project, and for individuals responding in more than 21 languages. I analyze data on 17 cultures from this project in order to get some insights on how cultures differ in their sentiments and how sentiments about some concepts vary across cultures. An affective map of the cultures derived with multi-dimensional scaling revealed that affective similarities and differences among cultures cannot be explained in terms of geography, nationality, or major religions. Underlying dimensions of the affective map perhaps relate to secularization and to a history of slavery/colonization. Meanwhile, sentiments about most concepts are remarkably similar across cultures, compared to the divergences of sentiments about different concepts. Thus, ubiquitous breakdowns in inter-cultural understandings must emerge from relatively small variations in feelings, or from issues where there are major differences in sentiments across cultures.

The International Academy for Intercultural Research is recognizing and celebrating early contributors to intercultural research, and University of Illinois psychologist Charles Osgood (1916 – 1991) was one of the most illustrious of these pioneers. A full biography is available elsewhere (Tzeng, 1990). Here I want only to observe that Osgood organized and managed the largest in depth cross-cultural study of the 20th century. That monumental study demonstrated beyond any doubt that affective meaning varies along three dimensions, within all 30 cultures considered in the project, for individuals responding in more than 21 indigenous languages.

Heise (2007), pp. 7–8 characterized the three dimensions as follows.

Sentiments have three aspects. Evaluation concerns goodness versus badness, Potency concerns powerfulness versus powerlessness, and Activity concerns liveliness versus quietness. The three aspects are abbreviated EPA. Each aspect, or dimension, of sentiments can be characterized by a variety of contrasts. [For example] some words characterizing the positive side of the Evaluation dimension are: nice, sweet, heavenly, good, mild, happy, fine, clean. Corresponding words for the negative side are: awful, sour, hellish, bad, harsh, sad, course, dirty. … Characterizations within each dimension are correlated. For example, something judged sweet is likely to be judged clean also. Characterizations across dimensions are uncorrelated. For example, sensing that something is powerful provides no clue as to whether it is good or bad.

These three basic dimensions of affective meaning were verified in each of 21 communities around the world as follows. First, researchers obtained indigenes’ adjective associations to 100 concepts that were familiar in all of the communities—concepts like *mother*, *danger*, and *thunder*. The second step was to have indigenes in each of the communities pair the adjectives that were acquired in the first step with opposites—e.g., *good* with *bad*, thereby forming end-points for 50 rating scales. In the third step 200 indigenous teenaged males rated the 100 concepts on the 50 scales. The ratings of the different respondents were averaged to get a single rating on each scale for each concept.

The final step involved factor analyzing the averaged ratings pan-culturally. That is, 50 scales in 21 cultures provided a total of 1050 variables. These variables were correlated across the 100 concepts, and the correlation matrix was factor analyzed in order to see if the first three factors were recognizably the Evaluation, Potency, and Activity dimensions that had been discovered in previous work within American culture (Osgood, Suci, and Tannenbaum, 1957). The second goal was to see whether every culture had scales loading on all three dimensions, indicating that every community made judgments along the same three dimensions as applied in other communities.

I forego further details about this monumental study because all that is necessary here is to emphasize that every precaution was taken to make sure that emergence of these dimensions in the different cultures was not a function merely of translating scales from English. Complete details on the study are provided in the book by Osgood, May, and Miron (1975), and a summary of the study is provided by Heise (2010).

Researchers in each community used twelve of the scales from the pan-cultural study—four scales for each EPA dimension—to assess the affective meanings of 620 concepts, with 40 teenaged males providing ratings of each concept. The intention of this part of the study was to create a cross-cultural Atlas that would provide a rigorous basis for cross-cultural analyses of affective meanings. Moreover, by the end of the project, data on the 620 concepts were collected in nine additional cultures beyond the 21 cultures included in the pan-cultural analyses.

## The atlas dataset

A few years after the publication of the Osgood et al. (1975) book, Charles Osgood was struck with a mentally incapacitating disease, and the project center on the University of Illinois campus disorganized. Data collected in the pan-cultural study, including the Atlas data, all were lost.

Fortunately, in 1978 at the University of Illinois bookstore I purchased a computer printout of results from the project containing results for 17 of the 30 cultures. I kept the printout in my files until I retired from teaching in 2001. At that point I scanned the printout sheets into electronic form, and I used optical character recognition technology to digitize the Atlas tables giving the mean ratings of 620 concepts by indigenous male teenagers in the 17 cultures. These data are the materials that I analyze in this talk^a^.

There are pros and cons in using the remnants of the cross-cultural Atlas for contemporary research. On one hand, the data are 50 years old, so findings may be out of date about specific cultures. Countering this consideration, the data were collected before globalization, and therefore these data give a unique picture of diversity in cross-cultural affective meanings before extensive intercontinental business, travel, and electronics shrank the world.

Another factor is that data loss cripples the overall dataset substantially. Data were lost from European cultures (Belgium, Finland, France, Greece, Italy, Sweden, and Hungary) and data also were lost from important Asian cultures (China, Japan, and two sites in Afghanistan). On the other hand, the data that still exist cover a variety of cultural communities worldwide: American Whites and American Blacks, Germans, Dutch, Yugoslavians, Turks, Iranians, Lebanese, Israelis, Indians from both New Delhi and Calcutta, Thais, Malays, Mexicans from Mexico City and Yucatan, Costa Ricans, and Brazilians.

Another weakness of the Atlas data is that ratings were obtained for only 620 concepts in each culture, whereas contemporary surveys using the EPA system for measuring affective meanings deal with 1500 to 3000 concepts (Heise, 2010). However, the contemporary surveys deal only with concepts relating to social interaction—role identities, interpersonal behaviors, social settings, and personal modifiers. The Atlas was designed to assess affective meanings for a broader variety of concepts relating to time, kinship, abstract symbolism, concrete symbolisms, environmental matters, carnalities, human activity, interpersonal relations, society, communications, philosophy, and ordinary things and stuff.

Finally, the Atlas data have missing data in some cultures, occasionally because a concept was inadvertently dropped, but usually because some concepts were too inflammatory to present in certain cultures. Thus, for example, *homosexual* was not rated in seven societies. On the other hand, there is no missing data at all for 532 concepts, and ratings were obtained in all but one or two communities for 606 concepts.

### Measuring distances

In this study I assess intercultural differences by measuring how far apart affective meanings are for each pair of cultures. Computing distance involves two steps. First, we measure how far apart the two communities are in their affective meaning of each particular concept. Second, we combine the distances for particular concepts into an overall distance between the two cultures.

**Figure 1:**
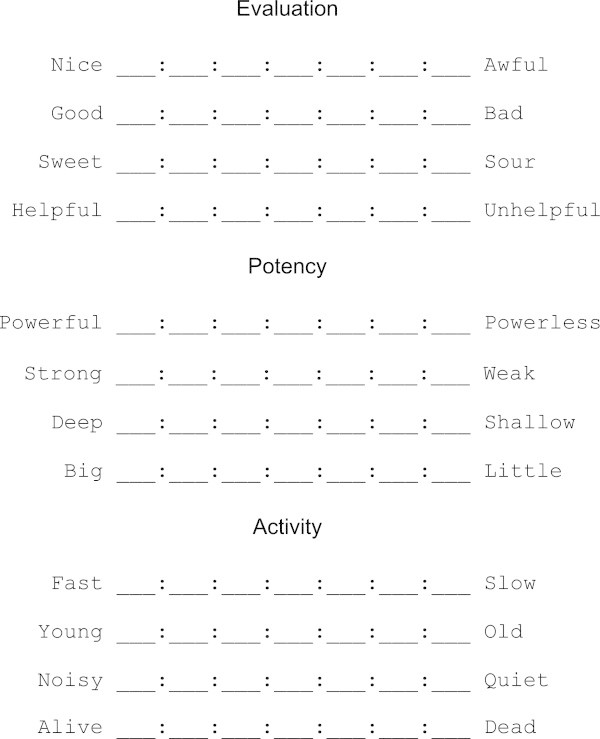
**Rating scales used to obtain data among White Americans.** Scales in other cultures were derived independently within the culture’s indigenous language.

Measuring meaning distances is possible because respondents rated concepts on EPA scales like the ones displayed in Figure 1. (Figure 1 shows the White-American scales that were used in the Atlas study). Ratings at the seven positions on each scale were coded as follows. The middle, or *neutral*, position was coded 0. Positions one place out from the middle, representing the adverb *slightly*, were coded +1 or -1: plus if on the nice, powerful, or fast side, and minus if on the awful, powerless, or slow side. Positions two places out from the middle, representing the adverb *quite*, were coded +2 or -2. Positions three places out from the middle, representing the adverb *extremely*, were coded +3 or -3. Ratings on the four scales for a particular dimension where averaged to get a respondent’s positioning of a concept on that dimension, and the average ratings for all 40 respondents where averaged to get the culture’s positioning of the concept on that dimension.

Each concept has three numbers attached to it—an EPA profile, with each number varying from −3 to +3. Minus values represent awfulness, powerlessness, or inactivity; and positive values represent niceness, powerfulness, or activity.

The distance between two concepts could be obtained by summing the absolute differences between the ratings on the three dimensions. Suppose, for example, that one concept is rated as slightly nice (+1), quite potent (2.0), and slightly inactive (−1.0). And suppose that another concept is rated as quite awful (−2.0), between neutral and slightly potent (0.5), and slightly active (1.0). Then the difference in affective meanings of the concepts can be computed as ((1) – (−2)) + ((2) – (0.5)) + ((1) – (−1)), or 6.5. However, absolute differences do not provide a measure corresponding to ordinary geometric distances, so instead I used Euclidean distances, which require squaring each of the differences before summing, and then taking the square root of the sum. (The Euclidean distance for this problem is 3.9).

To get the total distance between two cultures, I squared the Euclidean distance between the two cultures for each septe concept, summed the squares across all concepts^b^, and used the result as a measure of overall cultural distance in affective meaning. For example, this procedure showed that American Whites and American Blacks have a distance of 33.57. Meanwhile American Whites and Germans have an affective cultural distance of 32.8. American Blacks and Germans have a distance of 43.99.

### Multidimensional scaling

I conducted a nonmetric multidimensional scaling of the data, trying to reproduce the distances between cultures as distances in a physical space. For example, could the 17 cultures be positioned in a room-like space so that cultures very distant in their affective meanings also are very distant in the three-dimensional space of the room? Or could the distances between cultures be reproduced on a two-dimensional space, like a sheet of paper? Three and two dimensional spaces are useful for visualizing distances, but in principle reproducing cultural distances might require higher dimensional spaces that cannot be visualized easily.

A quantity produced during the scaling process called “stress” measures how well the inferred distances match the empirical distances, and this quantity provides guidance about how many dimensions are necessary. In this problem, if we try to reproduce all of the distances between cultures in a one-dimensional space (that is, along a line), the stress of the solution is a large value of 0.32. A two-dimensional solution reduces stress considerably to about 0.13. A three-dimensional solution reduces stress somewhat more to 0.07. The sequence of stress values for solutions with more than three dimensions declines slowly in a manner suggesting that the extra dimensions only are accommodating random variations. Thus, the solution in this case is either two-dimensional or three-dimensional.

**Figure 2:**
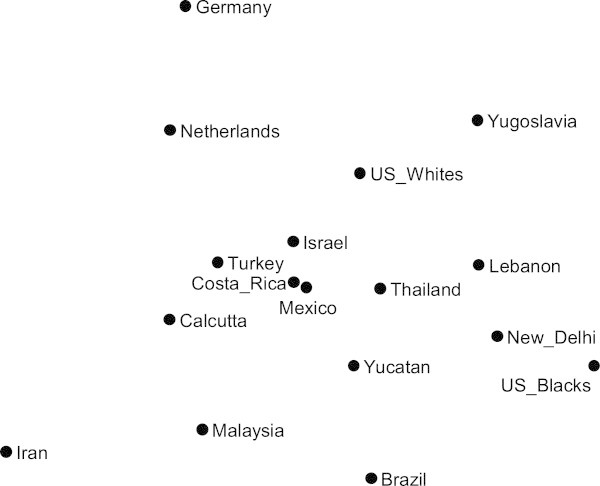
**Results of a nonmetric multidimensional scaling.** The chart shows cultures’ overall distances in affective meanings, in a two-dimensional representation.

Comparing the two and three dimensional solutions reveals that the three-dimensional solution is essentially the same as the two-dimensional solution except that Brazil projects out on a dimension of its own. In the two-dimensional solution Brazil is positioned at an extreme edge of the diagram in order to represent its divergence from other cultures (as can be seen in Figure 2). This report uses the two-dimensional solution for further analyses, both because it provides a reasonable representation of the empirical distances and because a two-dimensional solution is easy to visualize.

### Possible explanations

Figure 2 shows the distribution of the 17 cultures in a representation where physical distances correspond to cultural distances in affective meanings. For example, Yugoslavia and Iran are very far apart on the graph, corresponding to the fact that their affective meanings are very different. Germany and Brazil are far apart physically, corresponding to the fact that they, too, are far apart in affective meanings. Mexico City and Costa Rica are close on the graph, corresponding to the fact that affective meanings in these two cultures are similar.

Figure 2 shows the basic configuration of cultural distances between all 17 communities, and thereby can serve as the basis for testing possible explanations of why cultures are similar or dissimilar.

A first hypothesis derives from the fact that cultures in the Human Relations Area Files (HRAF, 2008) are organized by regions and sub-regions. This may be largely an administrative convenience, but it raises the question of whether geographic propinquity is a factor that explains cultural similarity. The hypothesis garners some support in Figure 2 in that the three topmost cultures are European (Germany, Netherlands, and Yugoslavia). Of course, many European cultures in the Atlas were lost so we cannot see whether such clustering would remain if Belgium, Finland, France, Greece, Italy, Sweden, and Hungary also were mapped. In any case, cultures representing other geographic regions do not cluster. Middle Eastern cultures span the width of the graph from Iran on one side, through Turkey and Israel, to Lebanon on the other side. Asian cultures—Calcutta India, Thailand, New Delhi India, and Malaysia—are similarly spread out even if not as extremely as the Middle Eastern cultures. New World cultures—U.S. Whites, Costa Rica, Mexico City, Yucatán, Brazil and U.S. Blacks—range up and down on the chart and from side to side. Thus, Figure 2 offers no convincing evidence that cultural similarity corresponds to co-presence in geographic regions.

A related hypothesis is that cultures within the same nation are more similar than cultures of different nations. This is testable because in three cases we have measurements from multiple communities within a single nation. The hypothesis does not fare well. The cultures of U.S. Whites and of U.S. Blacks are septed by a greater distance than septes the cultures of many nations. Similarly the cultures maintained in New Delhi and Calcutta India are more distant than many national cultures are. The two cultures measured in Mexico—Mexico City and Yucatán—are closer together than many other culture pairs, but even here the two are far from super-imposed. Thus the hypothesis that nationality determines culture may be rejected.

Major religions influence sentiments about some concepts so another hypothesis worth checking is that societies with the same predominating religion are culturally similar. However the cultures in predominantly Christian societies (Germany, Netherlands, U.S. Whites, Costa Rica, Mexico City, Yucatán, U.S. Blacks, and Brazil) are spread from top to bottom in Figure 2. Muslim cultures (Iran, Turkey, Malaysia, and Lebanon) are spread across the diagram. The two Hindu communities (Calcutta and New Delhi India) are across the diagram from one another. (Three other cultures – Israel, Thailand, and Yugoslavia – are single representatives of Judaism, Buddhism, and communism respectively.) Thus there is not much support for a religion connection to cultural similarity.

### Implicit dimensions

Yet intuitively Figure 2 does seem to have some substantive structure. Suppose that the graph were rotated so that the horizontal dimension passed from Iran to Yugoslavia. Then the horizontal axis might be viewed as something like ecclesiasticism versus secularism. That is, the communist society of Yugoslavia in the mid-20^th^ century represents the epitome of secularism, and Iran currently is an extreme ecclesiastical society (though the ecclesiastical structure was only imminent in pre-revolutionary Iran at mid-20^th^ Century). This interpretation of the rotated axis is strengthened by examining some of the specific concepts that most differ in Iran versus Yugoslavia. For example, large evaluation differences of 2.5 or more occur for *Prophet, Capitalism, God, Growing, Blood, Religion, King, Law, Prayer, Hospital, Tooth,* and *Homosexual*, with Iran more positive for all of these concepts. A third of these concepts relate to religion. Differences in affective meanings also emerge on the Activity dimension, with Iran usually attributing more activity to concepts.

Rotating the graph in order to make the horizontal dimension correspond to secularism versus ecclesiasticism raises the issue of what the vertical dimension would be in such a case. The vertical dimension, being perpendicular to the horizontal dimension, should represent something uncorrelated with secularism versus ecclesiasticism. The contrasting cultures in this case are Germany versus U.S. Blacks. Specific concepts that most differentiate these two cultures include the following: *Debt, Fighting, Baldness, Being Aggressive, Envy, Wine, Graft, Lying, Competition, Anger, November, Hunger, Bride, Defeat, Caste, Fear,* and *Sickness*. U.S. Blacks rated all of these except *Wine* and *Bride* less negatively on the Evaluation dimension than did Germans. U.S. Blacks also attributed lower activity to *Fighting, Being aggressive, Earthquake, Machine, Gramophone, Thunder, City, Youth, Jazz music, Adolescence, Play,* and *Boy*. Speculating, U.S. Blacks compared to Germans seem more accepting of emotions and consequences of social oppression; plus U.S. Blacks compared to Germans seem inclined to stay inactive or “cool” with regard to aggression and youth, which might be a wise strategy for an oppressed group. Such interpretations accord with previous discussions of the data from U.S. Blacks (Landis, McGrew, Day, Savage, and Saral, 1976;Sewell and Heise, 2010). Thus a possible interpretation of the second dimension is that it relates to a history of slavery and colonialism, with the controllers developing one set of affective meanings and the controlled developing another set.

### Distances for individual concepts

The Atlas data provide abundant opportunities to examine cultural differences in the affective meanings of specific concepts. I examine some of these differences in this section as a way of further elucidating cultural similarities and differences.

**Figure 3:**
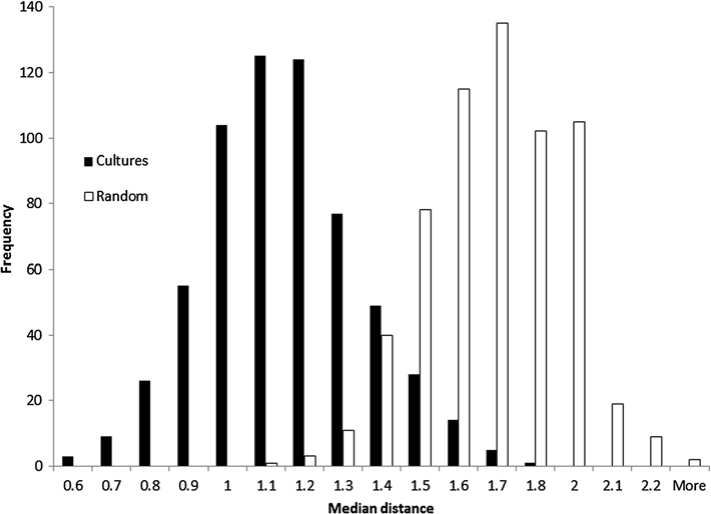
**Distributions of median inter-cultural distances in affective meanings of 620 concepts.** Each median is based on 136 pairs of cultures. Black bars show the distribution among real cultures. White bars show the distribution among randomly-constructed cultures.

A set of distances was computed for all 136 pairs of the seventeen Atlas cultures, for each of the 620 concepts in the Atlas. The median of the 136 distances for each concept is the statistic of interest here. Figure 3 displays a histogram showing the number of concepts at each interval of median distance. The bell shape of the distribution indicates that median distances between cultures’ affective meanings for concepts are small for some concepts (e.g., *Person*, *Stranger*, and *Neutrality*) and large for some other concepts (e.g., *Creature, Army*, and *Wine*), but mainly they are moderate in size (the rise in the middle).

Frequencies of concepts peak at the median distances of 1.1 and 1.2. That indicates that the typical difference between cultures in the affective meaning of a concept is a bit more than the difference between rating something as slightly good (a value of +1) versus quite good (+2), or quite powerful (+2) versus extremely powerful (+3), or slightly inactive (−1) versus neither inactive or active (0). Of course, maximum distances between Atlas cultures in the affective meanings of concepts are larger, up to 5.0 (which is the distance between Germany and Malaysia for the concept of “wine”). Nevertheless, the result for median distances does seem to indicate that cultural differences in sentiments are small.

Figure 3 also shows distances among imaginary cultures created with random affective meanings, for comparison with the distances among real cultures. The random results were obtained as follows. Within each EPA dimension and within each culture, the 620 mean ratings served as a population for drawing random values to assign to concepts. For example, German mean evaluations of the 620 concepts (ranging from −2.8 to +2.8 with a mean value of 0.77) served as a population of numbers from which new evaluations of concepts were drawn. To take a particular instance, the original mean evaluation of wine by German respondents was 2.3, and in the imaginary culture based on German EPAs this was replaced with 1.5, which was the mean evaluation by German respondents of some other concept. This method of randomization assured realistic random EPA values corresponding to actual EPA values within each culture. Seventeen imaginary cultures were created this way, each having EPA ratings generated randomly from one of the 17 actual cultures. The imaginary cultures served as the basis for re-computing median distances, with the results displayed in Figure 3.

Figure 3 shows that the distribution of distances among imaginary cultures with random affective meanings shifts to the right, indicating that random meanings are further apart on average than affective meanings within real cultures. This reinforces the conclusion that cultures have similar affective meanings for most concepts.

**Figure 4:**
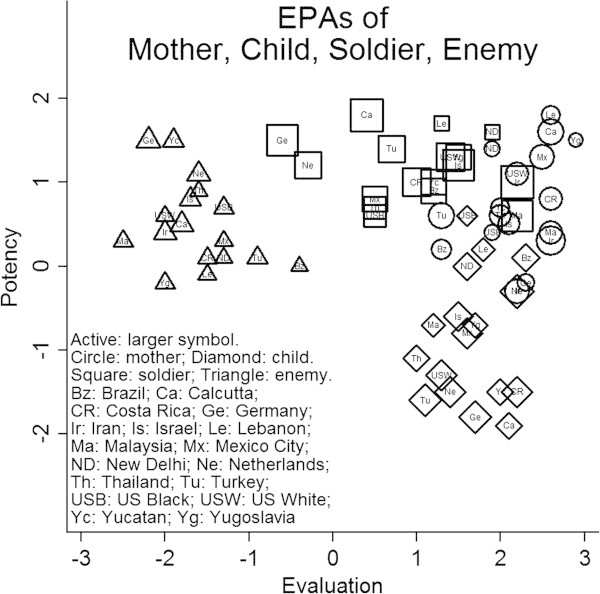
**Cross-cultural variations in affective meanings of Mother, Child, Soldier, and Enemy.** The graphic shows that cross-cultural variations often are less than inter-concept variations.

Figure 4 shows cross-cultural variations for four concepts with distances in the intermediate range– *Mother, Child, Soldier,* and *Enemy*. Having all four concepts on the same graph demonstrates that intra-concept variations across cultures are less than the inter-concept variations in the average position of concepts. For example, Evaluation and Potency ratings of *Enemy* vary cross-culturally, and Evaluation and Potency ratings of *Soldier* also vary cross-culturally, but ellipsoids enclosing the average ratings of all 17 cultures for each concept do not intersect. Similarly, in all cultures *Mother* and *Child* are felt to be good while differing substantially in potency. An ellipsoid around all the cultural meanings of *Mother* and another ellipsoid around all the cultural meanings of *Child* intersect some, but the two ellipsoids are mostly distinct. Among these four concepts, the affective meanings of *Soldier* and *Mother* are most intermingled on Evaluation and Potency, yet *Soldier* typically is more active than *Mother* (as indicated in Figure 3 by the larger symbols for *Soldier*) so ellipsoids for the two concepts would intersect only a little in the three-dimensional space.

Such findings lead to the speculation that affective meanings of particular concepts are largely the same cross-culturally, compared to differences in affective meanings between concepts. Evaluation ratings should correlate from one culture to another when computed across the 620 concepts. Similarly, Potency ratings of the 620 concepts should correlate across cultures, and Activity ratings should correlate across cultures. If within-dimension ratings of the 620 concepts correlate in all 17 cultures, then a single factor should emerge when the 17-by-17 matrix of inter-cultural correlations is factor analyzed. That is, a factor analysis of cross-cultural correlations of Evaluation ratings should be characterized by a single dominant factor, and the same should be true for Potency and Activity.

**Figure 5:**
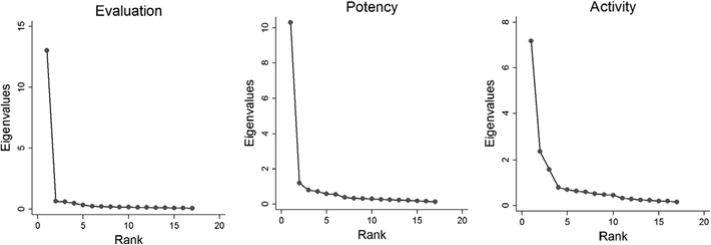
**Cross-cultural similarity in Evaluation, Potency, and Activity ratings of concepts.** The graphs show the sizes of each successive factor when correlations in affective meanings between the 17 cultures are factor analyzed. Affective meanings of different concepts are correlated across cultures to the extent that the first factor is much larger than later factors.

Figure 5 shows the size of each successive factor when the correlations between the 17 cultures are factor analyzed^c^. These results confirm the hypothesis of intercultural similarity in affective meanings for Evaluation and Potency. That is, Evaluations and Potencies of different concepts are correlated across all cultures. A dominant factor also is evident in the case of Activity, although the diagram shows that two additional factors also contribute to cross-cultural Activity correlations, indicating that there are clusters of cultures with higher Activity correlations within each cluster than between the clusters^d^.

To summarize, intercultural differences in affective meaning are very large for a few concepts and very small for a few concepts, while the vast majority of intercultural differences in affective meanings are moderate. Even the moderate intercultural differences in affective meaning are relatively small compared to differences in affective meaning for different concepts. The implication is that affective meanings are shared across cultures to a large extent, and a series of factor analyses of intercultural correlations within each of the EPA dimensions revealed considerable pllelism.

### Significance of cross-cultural differences

Correlations among cultures’ affective meanings make one wonder whether the majority of cultural differences in affective meaning really matter. This issue might be examined empirically by observing an equivalent interaction in different cultures to see if actions and reactions and accompanying emotions vary in any notable ways. For example, we might try to see if merchant-customer interactions are distinctive in different cultures.

An empirical cross-cultural study of this kind would be costly, but social psychology models are advanced enough at this point so that we can examine the issue through computer simulations. In particular, a program called *Interact* associated with affect control theory can be applied.

The basic idea in affect control theory is that humans try to experience the familiar. On the cognitive side, that means trying to fit new experiences into culturally available categories. On the affective side that is the focus of affect control theory, the principal means matching feelings experienced in actual situations with culturally normative sentiments. In particular, individuals try to design their actions so that their actions will produce feelings affirming cultural sentiments. A variety of studies have provided empirical support for affect control theory (Heise, 2013;Heise and Lerner, 2006;Heise and Weir, 1999;Schröder and Scholl, 2009;Smith-Lovin and Douglass, 1992;Wiggins and Heise, 1987), and various authorities have commended the theory (Clore and Pappas, 2007;Fararo, 1989;Kemper, 1991;Scholl, 2013). A detailed presentation of affect control theory is provided by Heise (2007).

Affect control theory employs a mathematical model grounded in quantitative measurements on the EPA dimensions, and the computer program *Interact* takes care of the mathematical analyses. Analyzing a social situation with *Interact* begins by specifying the identities of interactants. The program translates the identities into EPA profiles that the interactants try to confirm, computes the EPA profile for expected behaviors between the characters, and translates the behavior profiles into named behaviors. Specifying that a behavior actually occurred causes *Interact* to provide a variety of information about the action and its possible consequences.

For example suppose that we want to analyze interaction between a father and a daughter. We tell *Interact* that two people with those identities are interacting, and *Interact* translates the identity specifications into culturally defined EPA sentiments^e^ for *father* (2.46 2.54 0.76) and *daughter* (1.47 -0.04 1.11). Then we ask, what would a father do to a daughter? *Interact* computes the EPA profile (2.19 2.37 0.35) for the behavior that would produce feelings about the two characters most matching the cultural sentiments applying to them, and reports that *reason with* is one such behavior. We ask *Interact* to implement *reason with*, and *Interact* reports how the father and daughter feel emotionally during this action, indicating emotions both with words and emotional expressions on computer-drawn faces. *Interact* also predicts what each of the characters might do next after the father reasons with daughter. Additionally *Interact* reports how the individuals might be reconceptualized in various ways as a result of the action. For example, how might we view a father who reasons with his daughter (*considerate, perceptive, forgiving*), or how might we view a daughter who is reasoned with by her father (*sensitive, sympathetic, modest*).

### Storekeeper-customer interaction

I used *Interact* to examine the first behavior in storekeeper-customer interactions within each of the 17 Atlas cultures, plus contemporary American culture assessed among males at Indiana University in 2004. Among the Atlas cultures, the EPA profile for *Storekeeper* was used for the storekeeper, and the customer’s identity was specified in terms of the EPA profile for *Most people*. The Indiana analysis used the identities of *Merchant* and *Customer*. Table 1 gives the EPA profiles of each identity in the 18 cultures.

**Table 1:** **Evaluation, potency, and activity ratings of** ***storekeeper*** **and** ***most people*** **in 17 cultures (merchant and customer for 2004 U.S. Whites)**

	Storekeeper			Most people		
Culture	E	P	A	E	P	A
Brazil	1.2	0.5	0.4	1.0	0.4	0.6
Calcutta	0.1	0.6	1.1	0.2	0.6	0.7
2004 U.S. Whites	1.0	0.9	1.0	1.4	1.5	0.9
Costa Rica	1.1	0.5	0.5	1.2	0.3	0.8
Germany	0.9	0.8	1.1	0.7	0.4	0.7
Iran	1.3	0.0	1.4	1.2	0.5	1.5
Israel	0.3	−0.3	−0.1	0.1	0.5	0.0
Lebanon	0.9	0.4	0.4	0.2	0.3	0.1
Malaysia	1.8	0.3	1.2	1.0	0.3	0.6
Mexico City	0.9	0.8	0.6	1.0	0.5	0.7
Netherlands	0.8	0.1	0.3	−0.2	0.7	0.2
New Delhi	1.1	0.7	−0.5	1.2	0.6	0.0
Thailand	0.8	0.8	0.0	0.9	0.5	−0.1
Turkey	0.5	0.3	0.8	0.5	0.2	0.2
U.S. Blacks	1.0	0.6	−0.4	1.3	0.7	−0.1
U.S. Whites	1.3	0.3	0.3	0.9	0.3	0.9
Yucatan	1.9	0.7	0.5	1.5	1.3	1.0
Yugoslavia	−0.8	−0.6	0.0	1.1	1.3	−0.2

*Interact* derived the EPA profile for the optimal behavior of merchant to customer in each culture, and implementation of this action allowed *Interact* to compute interactants’ accompanying emotions^f^. K-means clustering applied to the EPA profiles of the interactants’ identities, the EPA profiles of the predicted first action, and the interactants’ emotion EPA profiles during the first action reduced the 18 sets of results to five different patterns.

Table 2 lists the cultures in each cluster. Table 2 also shows the mean EPA profiles for optimal behaviors, storekeeper emotions, and customer emotions, averaged over all of the cultures in a cluster.

**Table 2:** **Mean evaluation, potency, and activity values for predicted behaviors and emotions, averaged across cultures within each k-means cluster**

Cluster	Behavior			Storekeeper emotion			Customer emotion		
	E	P	A	E	P	A	E	P	A
Iran, Malaysia, 2004 U.S. Whites, Yucatan	1.56	0.17	0.75	1.91	0.18	1.04	1.42	0.37	0.61
New Delhi, Thailand, U.S. Blacks	1.20	0.51	−0.46	1.58	0.53	0.46	1.21	−0.30	0.63
Brazil, Costa Rica, Germany, Mexico City, U.S. Whites	1.20	0.39	0.26	1.55	0.46	0.87	1.25	−0.08	0.63
Calcutta, Israel, Lebanon, Netherlands, Turkey	0.61	0.11	0.17	0.99	0.24	0.86	0.93	−0.24	0.53
Yugoslavia	0.10	−0.72	0.13	1.21	−0.51	0.80	0.89	−0.40	0.52

**Figure 6:**
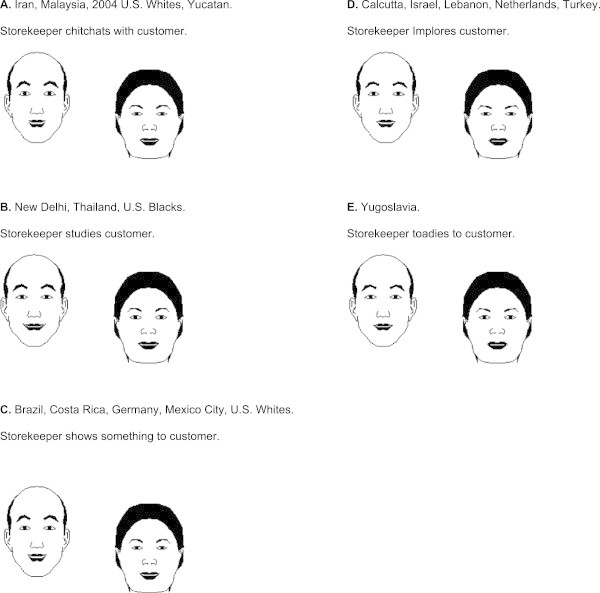
**Simulations of a storekeeper-customer encounter.** Panels **A** through **E** each represents a cluster of cultures that is largely homogeneous with regard to simulated behaviors and emotions arising in a storekeeper-customer encounter. The displayed behaviors and emotions, based on EPA profiles presented in Table 2, were obtained with software for simulating social interactions.

Figure 6 also lists the cultures in each cluster, and expands the numerical results in Table 2 by offering a verbal description of the first action of storekeeper to customer within each cluster. The verbal behavior was obtained by finding a named behavior whose EPA profile among Indiana males in 2004 was close to the mean profile for the optimal behavior in a cluster. The description of the storekeeper’s action is inexact since it is not based on behavior measurements made in the cultures within a cluster, but the description does provide some sense of how the first action varies from one cluster to another.

Additionally Figure 6 presents the computer-drawn facial expressions of storekeeper and customer during the first action of their encounter. The facial expressions were drawn by *Interact* from the mean EPA profiles for emotions given in Table 2. The mean EPA profiles for behaviors, storekeeper emotions, and customer emotions within the five clusters are provided in Table 2.

Examining Figure 6, we see that predicted behaviors in all clusters are plausible ways that a merchant might engage a customer, but the nature of the interaction varies cross culturally. In cluster A (upper left), the relationship is egalitarian. In cluster B (next down) the merchant is quietly attentive. In cluster C (lower left) the merchant is businesslike. In cluster D (upper right) the merchant is supplicating. In Cluster E, comprised of only Yugoslavia, (lower right) the merchant acts in a servile manner. Facial expressions of emotion in clusters A through D indicate mutually engaged parties having a pleasant encounter. In cluster B both storekeeper and customer appear reserved but positive. In E the encounter is somewhat strained. Overall, this analysis suggests that the merchant-customer relation might be realized cross culturally with at least five distinctive kinds of action, and three emotional tones.

Because of the substantial similarity in affective meanings of relevant concepts cross-culturally, travelers can accomplish commercial exchanges everywhere, and usually enjoy them, even though in some places the interaction may have a foreign quality, at variance with what one learned to expect at home. Nevertheless, variant forms of the relationship may have different consequences—e.g., some storekeeper-customer relationships may be more efficacious than others in terms of sales made, or customer satisfaction.

## Conclusions

Analyses of cross cultural data collected in the mid-20th century show that similarities and differences in cultures’ affective meanings are not based on geography, nationality, or religious creed. Cultures do vary along two major dimensions which seem to be secularism-versus-ecclesiasticism, and a dimension relating to colonialism and slavery that contrasts cultures of the controllers with cultures that emerged among the controlled.

Intercultural differences in affective meanings of specific concepts are large in a few cases, very small in a few cases, and moderate in the vast majority of cases. Cultures largely agree regarding the affective meanings of most concepts, at least in the sense that cultural differences for any specific concept are relatively small compared to average cross-cultural feelings about different concepts. Heise (2001) obtained similar results, using a correlational methodology instead of the distances computed here. This suggests that feelings about things have an ontological core that is largely shared across cultures—for example, war is bad, mothers are good, and children are weak. Future studies might profitably pursue this notion in studies of morality and ethics.

Observing that cultural differences are relatively small does not necessarily mean that they are irrelevant. A cross-cultural simulation analysis of the storekeeper-customer relationship indicated cross-cultural differences in behaviors and emotions within the relationship that probably have material consequences for this market mechanism in terms of its efficiency and intrinsic satisfaction. A similar analysis of mother-child relationships cross-culturally (not reported here) produced differences that might have important consequences in socializing individuals with various personality traits. The strategy introduced here of clustering data and simulation results may provide a path for studying such matters economically. Use sentiments data obtained in surveys to identify clusters of cultures, and study the phenomenon of interest ethnographically in a representative of each cultural cluster.

The data analyzed here were collected in the mid-20th century before large scale globalization. The convergence of affective meanings in the mid-20th century suggests that diversity already was limited before international business, travel, and electronic media shrank the world. Furthermore, data collected since the Atlas study (see Heise, 2010) suggest that as much diversity in affective meanings exists in the globalized world as in the pre-globalized world.

Overall we have found in this study that something about a given concept makes people everywhere tend to converge on a more-or-less shared affective meaning for the concept, but other somethings impel different groups to develop their own slant with regard to the affective meaning of that concept. This is true in the globalized world as much as it was true in the pre-globalized world.

## Endnotes

^a^Please download the photocopies of the printouts and the digitized files of the Atlas data so that there is no chance of losing these valuable data in the future. Practically none of the potential of the data has been tapped. Osgood et al. (1975) systematically analyzed only colors, and other reports based on the data dealt with a specific culture (e.g., Landis, McGrew, Day, Savage, and Saral, 1976). Thus the data still can serve as a basis for myriad future analyses. The URL at which you will find the materials for download is http://www.indiana.edu/~socpsy/Atlas/.

^b^The summations were over all 620 concepts, with missing data values imputed as the average distance among all cultures with empirical data for the given concept. I also computed the distances with listwise deletion of cases with missing data, and obtained essentially the same results.

^c^Technically, Figure 4 shows scree diagrams of the eigenvalues resulting from Q-method component analyses of Evaluation scores, Potency scores, and Activity scores.

Correlations between cultures’ Evaluations ranged from 0.38 to 0.90, with all but one in the range 0.51 to 0.90. Correlations between cultures’ Potency means ranged from 0.24 to 0.82. Activity correlations ranged from −0.18 to 0.80, with 70 percent of the correlations having a value of 0.20 or higher. Missing data was handled in the correlation analyses by listwise deletion, giving 535 concepts for Evaluation and Activity, and 533 for Potency.

Lack of completely smooth curves after the first eigenvalues in Figure 4 suggests that some clustering of cultures also occurs with regard to Evaluation and Potency scores. On Evaluation: a component groups Germany, Israel, Netherlands, U.S. Blacks, Yugoslavia; another groups Iran, Lebanon, New Delhi, Calcutta, Malaysia; and a third contrasts U.S. Blacks and Brazil. On Potency: a component groups Germany and the Netherlands; and another groups Yugoslavia and Lebanon. On Activity: a component groups Turkey, Iran, Calcutta, Malaysia, Mexico, Costa Rica; another groups Yugoslavia, Lebanon, New Delhi, Thailand; and a third groups U.S. Blacks, Germany, Netherlands, Israel, Brazil. A major study is needed to examine such cultural clusters and the concepts contributing to them.

^d^This example uses sentiment measurements obtained from undergraduate males in Indiana, 2002–2004.

^e^These analyses used Atlas EPA profiles for Storekeeper and Most People. EPA profiles for behaviors and emotions were derived with impression-formation equations estimated in a U.S.A. study. Thus the basic data are indigenous, but predictions derived from the data involve non-indigenous equations which might introduce some errors into the predictions.
